# The Italian ICD-11 field trial: clinical utility of diagnostic guidelines for schizophrenia and related disorders

**DOI:** 10.1186/s13033-020-0338-z

**Published:** 2020-01-24

**Authors:** Mario Luciano, Gaia Sampogna, Valeria Del Vecchio, Vincenzo Giallonardo, Carmela Palummo, Benedetta Pocai, Luca Steardo, Francesca Zinno, Tahilia Rebello, Geoffrey M. Reed, Andrea Fiorillo

**Affiliations:** 1 WHO Collaborating Center for Research and Training in Mental Health, University of Campania “L. Vanvitelli”, Naples, Italy; 2 Dipartimento di Scienze della Salute, Università della Magna Graecia, Catanzaro, Italy; 3 WHO Collaborating Centre for Capacity Building and Training in Global Mental Health, Department of Psychiatry, Columbia University Vagelos College of Physicians and Surgeons, New York, NY USA

**Keywords:** ICD-11, Clinical utility, Diagnosis, Schizophrenia, Schizoaffective disorder, Goodness of fit

## Abstract

**Background:**

The 11th revision of the International Classification of Diseases and Related Disorders (ICD-11) has been released. In order to test the clinical consistency and the clinical utility of the proposed guidelines the World Health Organization (WHO) has carried out the Ecological Implementation Field Studies in various countries. In this paper the results of the Italian field trials on the clinical utility of the ICD-11 diagnostic guideline concerning schizophrenia and related disorders will be presented.

**Methods:**

In Italy, field trials have been carried out at the Department of Psychiatry of the University of Campania “L. Vanvitelli”. All patients showing any psychotic symptom and referring to the outpatient and inpatient units have been recruited. Patients were interviewed by two clinicians with whom they had not had any prior clinical contact. At the end of each interview, clinicians were asked to complete 12 questions about the clinical utility of the diagnostic guidelines as applied to each patient.

**Results:**

Fourteen clinicians and 100 patients have been involved. The ICD-11 clinical guidelines were perceived as easy to use, with an adequate goodness of fit, clear and understandable and with an adequate level of details and specificity to describe the essential features of the diagnoses. Clinicians rated very positively their usefulness in describing the threshold between patient’s disorder and normality. Despite still very positive, the guidelines have been perceived as less useful to select a treatment, to assess patients’ prognosis and to communicate with other mental health professionals.

**Conclusions:**

The 11th revision of the chapter on Mental, Behavioural and Neurodevelopmental Disorders has made substantive changes to the conceptualization of mental disorders which could have impacted on their reliability and clinical utility. Results of the Italian field studies, in line with those reported by the international sample, highlight that ICD-11 has been rated as highly clinically useful by participating clinician, more than the ICD-10. This could be considered a good reason to be optimistic about the implementation of the ICD-11 among global clinicians.

*Trial registration* The study has been approved by the Ethical Review Board of the University of Campania “L. Vanvitelli” (N. 416, 2016)

## Background

The World Health Organization (WHO) periodically releases the International Classification of Diseases and Related Disorders (ICD) which represents the main instrument for the identification of health trends and statistics worldwide [45]. The ICD is a diagnostic classification system listing all disorders, diseases, injuries and other related health conditions and it is commonly used for monitoring the incidence and prevalence of diseases, analyzing reimbursements and resource allocation trends in national health systems, and for evaluating the quality of clinical guidelines [16].

The 11th revision of the International Classification of Diseases and Related Disorders (ICD-11) has been released to its member states on June 18, 2018 [2, 32]. The ICD-11 will now be translated into different languages and implemented in routine care starting from January 2022 [36]. The process of revision of the ICD has been primarily focused on the improvement of construct validity and clinical utility of diagnostic categories, while the development of the Diagnostic and Statistical Manual of mental disorders (DSM) has been mainly guided by concerns of construct validity [3, 11, 23, 31].

The clinical utility of a classification system is distinct from its validity, although these two constructs are highly correlated, since a diagnosis cannot have a good value in terms of clinical utility but lacking any clinical validity [17, 29, 42]. Ideally, a classification system with a satisfying level of clinical utility should allow clinicians to identify the best diagnostic category for each patient and should provide useful information on disease’s treatment and management [22, 28, 40].

Currently available diagnostic systems have been criticised for having a limited clinical utility [1, 5, 17, 27]. In fact, a high proportion of diagnoses of mental disorders are recorded as “Unspecified” (the term used in the ICD) or as “Not Otherwise Specified” (as in the definition of the Diagnostic and Statistics Manual of Mental disorders—DSM), suggesting that the boundaries between the different diagnostic categories are not so well defined. Furthermore, a significant proportion of people with mental disorders meet criteria for two or more mental disorders at the same time [20], which can be considered as an artefact of the current classification systems, rather than a true comorbidity issue [26, 41]. It may be that different disorders represent different aspects of the same underlying condition, or that the threshold for the diagnosis of some conditions which do qualify as mental disorders may be too low, so that also normal states are included [19, 26, 43, 46]. Finally, the clinical heterogeneity of the current diagnostic categories further underlines the lack of clinical validity [18]. The case of major depressive disorder (MDD) in the DSM-5 is paradigmatic, since two patients can receive the same diagnosis of MDD even without presenting any single symptom in common [6].

According to the WHO Department of Mental Health and Substance Abuse, the clinical utility of a classification construct or category for mental and behavioural disorders depends on: (a) value in communication (e.g., among practitioners, patients, families, administrators); (b) implementation in clinical practice, including goodness of fit (i.e., accuracy of description), ease of use, and time required to use it (i.e., feasibility); and c) usefulness in choosing interventions and making clinical decisions [34].

In order to improve the clinical utility of the revised version of the ICD, Clinical Descriptions and Diagnostic Guidelines (CDDG) have been developed with the aim to provide clinicians with clearly organized and consistent information across disorders. These guidelines can be adapted to the cultural background of clinicians and give them the possibility to use their clinical judgment [4]. Moreover, the global structure of the ICD-11 CDDG is consistent with the clinical routine practice [35, 37, 40].

The clinical utility and the diagnostic reliability of CDDGs have been tested in clinic-based field trials with the goal to improve clinical utility while maintaining diagnostic reliability [4, 12].

As part of the ICD-11 developmental field studies [36, 38], the WHO Collaborating Center for Research and Training in Mental Health at the Department of Psychiatry of the University of Campania “L. Vanvitelli” has been included in the multicentric study on the reliability and clinical utility of the ICD-11 CDDG. In this paper, we present the data on the clinical utility of the ICD-11 CDDG based on the Italian data.

## Methods

The clinical utility and reliability of the ICD-11 diagnostic guidelines have been assessed in two protocols implemented at 28 sites in 13 countries. Protocol 1 assessed the utility and reliability of the clinical guidelines for schizophrenia/other primary psychotic disorders and for mood disorders, while Protocol 2 tested the guidelines for mood disorders, anxiety and fear-related disorders, and disorders specifically associated with stress.

All patients showing any psychotic symptom and referring to the outpatient and inpatient units of the Department of Psychiatry of the University Vanvitelli of Naples, in the period from September 2016 to September 2017, have been asked to participate. Patients were excluded if they: (1) had difficulties in understanding due to a severe cognitive impairments (e.g., a confirmed neurodevelopmental or neurocognitive disorder); (2) were not fluent in the primary language of the local personnel; (3) suffered from current incapacitation due to severe physical illness or pain; (4) had current substance intoxication or withdrawal; (5) had current imminent risk of self-harm, danger to others, or serious medication side effects.

Eligible patients were provided with all relevant information on the study characteristics in order to collect their informed consent. Subsequently, patients were interviewed by two clinicians with whom they had not had any prior clinical contact. One clinician served as the primary interviewer and the second clinician as the observer, who could ask additional questions at the end of the interview. The clinician raters were instructed to run the joint-rater interview for about 60–90 min. They were asked to use the same approach as they would in their routine practice. Therefore the range and the length of the diagnostic interviews were substantially consistent with usual practice in participating mental health centers. Based on the interview, clinicians were allowed to formulate up to three diagnoses. Diagnoses were non-hierarchical (i.e., not specified as primary, secondary or tertiary) and could fall within any ICD-11 mental, behavioural or neurodevelopmental disorder diagnostic group. It was also possible to specify the presence of a non-mental or behavioural disorder, or even the absence of any mental or behavioural disturbance. Patients have not been interviewed by their referring or treating clinician in order to avoid any bias due to the previous knowledge of clinicians with the patient.

At the end of each interview, clinicians were asked to answer to 12 questions about the clinical utility of the diagnostic guidelines per each patient. In particular, questions addressed: (1) clinical utility (ease of use, goodness of fit, clarity and understandability); (2) implementation characteristics of the guidelines (level of detail, feasibility of assessment requirements, time required); (3) utility of specific sections of the guidelines (boundaries with normality and differential diagnosis); (4) utility of the guidelines for specific purposes (selecting treatments, predicting prognosis, communicating with other professionals, educating patients and family members). Average time to respond to the 12 clinical utility questions is about 15 min.

### Characteristics of the study site

Participating sites were chosen among the WHO Collaborating centres, which are designated by the Director General to carry out activities in support of the WHO’s programmes.

In Italy, the ICD-11 developmental field study, Protocol 1, has been implemented at the WHO Collaborating Center for Research and Training in Mental Health, Department of Psychiatry of the University of Campania “L. Vanvitelli” in Naples. The Department includes two inpatient units for voluntary and acute admissions; one day-hospital unit and several outpatient units for the management and treatment of mood disorders, psychotic disorders, anxiety disorders, eating disorders, cognitive psychotherapy, family treatment, psychosocial rehabilitation. The centre provides an average of 15.000 visits per year, with a mean number of 1.000 new patients per year. The staff of the Department includes 15 psychiatrists, 3 psychologists, 23 nurses, and 100 residents in psychiatry.

### Sample size calculation

The sample size have been determined by the WHO based on the total number of participating centres, based on the multicentric design of the study.

For the inter-rater reliability assessments, assuming an alpha level of .05 and power of .80, 53 participants per each centre are necessary to detect a kappa value of .4 (fair reliability), when the percentage of the target diagnosis is 20% of the patients referring to the centre. For rarer disorders with a percentage of 10%, 65 participants would be needed in order to detect the same effect size. Higher kappa values (e.g., .7 or good reliability) would require fewer participants (16 and 19, respectively, for percentages of 20% and 10%). These estimates assume kappa will be calculated separately for each diagnosis (i.e., k1 = the target diagnosis, k2 = all other diagnoses). Therefore each participating centre, has recruited 100 patients per each protocol. This number is adequate to calculate the inter-rater reliability of the main diagnoses for each sample, and for the global sample when data are taken together.

For the clinical utility study, the need is to have a reasonable number to provide meaningful frequency counts. For continuous variables, 10 participants are needed to achieve a power of .8 assuming an alpha of .05 (two-tailed) in order to detect one point of difference from the midpoint of the rating scale of an item (i.e., to detect a preference or non-preference) with an estimated standard deviation equivalent to one point on the scale. For discrete variables, 44 participants are necessary to detect an effect size of .5 (taken from similar questions in the ICD-11 case-controlled field trials) to achieve a power of .8 assuming an alpha of .05 (two-tailed). Therefore, for utility assessment for a selected indexed condition, a minimum of 50 patients recruited from at least two geographically distinct study sites will be required.

### Training to the ICD-11 clinical diagnostic guidelines

The study coordinator was responsible for clinicians’ recruitment. At the Italian site, participating clinicians were psychiatrists or advanced residents in psychiatry (i.e., trainees with more than 2 years of residency) and were qualified to make diagnoses of mental disorders as part of their clinical practice. Advanced residents in psychiatry have been included as interviewers, but they were always paired with senior clinicians. Participating clinicians received the ICD-11 diagnostic guidelines and were asked to read them prior to the training session. The training session, which was focused on the core features of the ICD-11 diagnostic guidelines covered by Protocol 1 and their differences with the ICD-10, was carried out using a standardized set of slides provided by the WHO. Interactive exercises were provided in order to familiarize with the guidelines on case vignettes. No other instruction was given on how to run the interview, which was left to the experience of clinician raters, according to their professional training and usual practice. The training session lasted for approximately 2 h. During the training session, information regarding the study flow and data collection procedures were provided. Following the 2-h training session and prior to the start of patients’ recruitment, clinicians were asked to register to the online registration platform, for collecting information on their main socio-demographic and professional characteristics.

At the Italian site, ICD-11 guidelines, training materials, and other study materials were used in the English version, while the clinical interviews were conducted in Italian language, in order to replicate the ordinary practice circumstances. Details on clinicians’ recruitment and training, study implementation processes, data collection, and ethical statements have been provided elsewhere [36, 38].

### Data collection

Data were entered by clinicians into the Electronic Field Study System (EFSS), a secure web-based data collection system developed using Qualtrics™ (Provo, UT, USA) survey software. Data were stored and managed centrally by the Data Coordinating Center (DCC) at Columbia University.

Data quality was guaranteed through continuous monitoring of data collection procedures by local research staff at each site and through use of programmed functions within Qualtrics™, such as forced response and content validation options, thus collecting data standardized and uniform format from all sites. Site-based research team performed data-check on a regular basis; in case of mistake, this has been forwarded to the DCC for data correction.

### Data analysis

Clinicians’ responses to each of the 12 clinical utility items were analysed using frequency counts for each response. To provide metrics of overall favourable responses, ratings of “Quite” and “Extremely” were collapsed. Responses to the clinical utility variables by country were also calculated (not reported here; available from the authors upon request).

For reliability analyses, intraclass kappa coefficients were calculated with bootstrapped 95% confidence intervals, based on 1000 resamples, for each country. Reliability coefficients were calculated only for the most common diagnoses in our sample (i.e., N ≥ 15), to maximize the chance of having a sufficient number of diagnoses to estimate kappa. Per-diagnosis ratings of clinical utility were also calculated for these same diagnoses.

## Results

### Clinicians’ and patients’ socio-demographic characteristics

Fourteen clinicians have been involved in the study in Italy. They were predominantly male (64.3%) with a mean age of 39.8 ± 6.2 years, psychiatrists (64%) or trainees in psychiatry (36%), and with an average of 7.7 ± 7.2 years of professional clinical experience.

One hundred patients have been recruited for the Protocol 1, with a mean age of 41.4 ± 11.2 years, mainly single (71%) and not employed (74%) (Table 1). Ten patients refused to participate in the study. Reasons for refusal included: (1) not interested (N. 4); (2) lack of time (N. 3); (3) organizational difficulties (N. 3). There were no differences between patients who refused to participate and those who completed the study.

**Table 1:** Patients’ socio-demographic characteristics (N = 100)

Age, years, M ± SD	41.4 ± 11.2
Gender, male N (%)	50 (50.0)
Relationship status, N (%)	
Single	71 (71.0)
Married/cohabitating	19 (19.0)
Separated/divorced	7 (7.0)
Widowed	3 (3.0)
Employment, N (%)	
Full time	11 (11.0)
Part time	9 (9.0)
Unemployed	74 (74.0)
Student	4 (4.0)
Retired	2 (2.0)
Treatment setting, outpatient N (%)	67 (67.0)

### Clinical utility ratings

Clinical utility ratings are shown in Table 2. Considering that both clinicians completed the clinical utility survey at the end of each interview, the expected number of completed assessment would be 200. We obtained 198 clinical utility ratings since two clinicians did not complete the survey after the interviews.

**Table 2:** Clinical utility questions and responses (N = 198)

*Core clinical utility questions*				
Please rate the overall ease of use of the diagnostic guidelines with respect to this patient:				
Not at all:	Somewhat:	Quite:	Extremely:	Quite + extremely:
0	13 (6.6%)	123 (62.1%)	62 (31.3%)	185 (93.4%)
Please rate the overall goodness of fit or accuracy of the diagnostic guidelines with respect to this patient:				
Not at all:	Somewhat:	Quite:	Extremely:	Quite + extremely:
0	11 (5.6%)	121 (61.1%)	66 (33.3%)	187 (94.4%)
Please rate the extent to which the diagnostic guidelines were clear and understandable overall as applied to this patient:				
Not at all:	Somewhat:	Quite:	Extremely:	Quite + extremely:
0	7 (3.5%)	113 (57.1%)	78 (39.4%)	191 (96.5%)
*Implementation characteristics*				
Which of the following statements best describes your evaluation of the level of detail and specificity of the essential features for the diagnosis or diagnoses that you applied to this patient?				
Insufficient:	About the right amount:	Too much:		
1 (0.5%)	158 (79.8%)	39 (19.7%)		
Please rate the extent to which the guidelines imposed assessment requirements that were difficult to apply to this patient (e.g., requirements that rely too much on the patient’s memory of remote events or the patient’s ability to report temporal relationships between symptoms):				
Very difficult:	Somewhat difficult:	Quite easy:	Extremely easy:	Quite + extremely easy:
1 (0.5%)	23 (11.6%)	121 (61.1%)	53 (26.8%)	174 (87.9%)
How would you describe the amount of time that it took you to apply all of the Essential Features to this patient for the diagnosis or diagnoses that you selected, in comparison to your usual clinical practice?				
Much longer:	Somewhat longer:	About the same:	Shorter:	
0	6 (3.0%)	150 (75.8%)	42 (21.2%)	
*Specific sections*				
Please rate the extent to which the description of the boundary between disorder and normality contained in the guidelines was useful as applied to this patient:				
Not at all:	Somewhat:	Quite:	Extremely:	Quite + extremely:
2 (1.0%)	19 (9.6%)	97 (49.0%)	80 (40.4%)	177 (89.4%)
Please rate the extent to which the description of the boundary between this patient’s disorder and other disorders (section on differential diagnosis) was useful as applied to this patient:				
Not at all:	Somewhat:	Quite:	Extremely:	Quite + extremely:
2 (1.0%)	14 (7.1%)	105 (53.0%)	77 (38.9%)	182 (91.9%)
*Specific uses*				
How useful would the diagnostic guidelines be in helping you to select a treatment for this patient?				
Not at all:	Somewhat:	Quite:	Extremely:	Quite + extremely:
5 (2.5%)	42 (21.2%)	112 (56.6%)	39 (19.7%)	151 (76.3%)
How useful would the diagnostic guidelines be in helping you to assess this patient’s prognosis?				
Not at all:	Somewhat:	Quite:	Extremely:	Quite + extremely:
3 (1.5%)	46 (23.2%)	107 (54.0%)	42 (21.2%)	149 (75.3%)
How useful would the diagnostic guidelines be in helping you to communicate about this patient with a colleague or other health care professional?				
Not at all:	Somewhat:	Quite:	Extremely:	Quite + extremely:
2 (1.0%)	48 (24.2%)	94 (47.5%)	54 (27.3%)	148 (74.7%)
How useful would the diagnostic guidelines be in helping you to educate this patient and/or family about his or her condition?				
Not at all:	Somewhat:	Quite:	Extremely:	Quite + extremely:
3 (1.5%)	51 (25.8%)	98 (49.5%)	46 (23.2%)	144 (72.7%)

Missing data: two clinicians did not complete the 12-items questionnaire at the end of the interview

As regards the core clinical utility questions, almost all clinicians were quite or extremely satisfied with the “Ease of use” (93.4%), the “Goodness of fit/accuracy of proposed diagnostic guidelines” (94.4%), and the “Overall understandability” of the CDDG (96.5%).

Concerning implementation characteristics, 79.8% of clinicians reported that the ICD-11 diagnostic guidelines have an adequate level of details and specificity to describe the essential features of the diagnoses. Moreover, 87.9% of clinicians considered the CDDG easy to use with patients, stating that the same (75.8%) or even less (21.2%) time is required to apply the guidelines in routine ordinary practice.

The majority of clinicians rated very positively the usefulness of CDDG in describing the threshold between patient’s disorder and normality (89.4%) and between patient’s disorder with other disorders (91.9%).

Furthermore, the CDDG were rated as extremely useful in selecting a treatment (76.3%), assessing patients’ prognosis (75.3%), communicating with colleagues or other health care professionals (74.7%) or in educating patients and/or family members about patient’s clinical condition (72.7%) (Table 2).

As regards concurrent reliability or joint rater agreement, the value of intraclass kappa for the diagnosis of “schizoaffective disorder” was 0.79, being lower than that for the diagnosis of “schizophrenia” (intraclass kappa = 0.85). However, both diagnoses were considered as understandable (Schizoaffective disorder: 99.2% vs. Schizophrenia: 100%) and easy to use (Schizoaffective disorder: 91.2% vs. Schizophrenia: 97.5%) (Tables 3 and 4).

**Table 3:** Concurrent reliability of diagnoses for diagnoses that were selected at least 15 times

	Number of diagnoses (N)	Joint rater agreement (intraclass kappa)	Standard error	Bootstrapped 95% CI
Schizophrenia	65	0.85	0.053	0.74–0.96
Schizoaffective disorder	20	0.79	0.087	0.59–0.93

**Table 4:** Clinical utility ratings for three core questions for diagnoses of disorders that were selected at least 15 times

	Not at all	Somewhat	Quite	Extremely	Quite + extremely
Ease of use					
Schizophrenia	0	3 (2.5%)	73 (60.3%)	45 (37.2%)	118 (97.5%)
Schizoaffective disorder	0	3 (8.8%)	25 (73.5%)	6 (17.6%)	31 (91.2%)
Goodness of fit					
Schizophrenia	0	3 (2.5%)	73 (60.3%)	45 (37.2%)	118 (97.5%)
Schizoaffective disorder	0	3 (8.8%)	23 (67.6%)	8 (23.5%)	31 (91.2%)
Clarity and understandability					
Schizophrenia	0	1 (0.8%)	67 (55.4%)	53 (43.8%)	120 (99.2%)
Schizoaffective disorder	0	0	22 (64.7%)	12 (35.3%)	34 (100.0%)

## Discussion

The 11th revision of the ICD required more than 10 years of intensive work and the involvement of hundreds of experts as members of the Advisory and Working Groups as consultants [25]. Moreover, the revision process has required an extensive collaboration with WHO member states, funding agencies, professional and scientific societies, and it has been defined as “the most global, multilingual, multidisciplinary and participative revision process ever implemented for a classification of mental disorders” [35], which included users’ perspective [8, 14], and cultural differences in the presentation of mental disorders [13]. The revision of the Chapter on Mental, Behavioural and Neurodevelopmental Disorders has brought significant changes to the conceptualization of many disorders, which may have an impact on their validity and clinical utility [2, 9].

Overall, findings from the Italian field trials are in line with those of the global international sample and confirm the perceived clinical utility of ICD-11. In particular, Italian clinicians found that the ICD-11 CDDG are easy to use, accurately detailed, with a good correspondence to patients’ clinical presentations (i.e., goodness of fit), clear and understandable. Furthermore, clinicians reported that the use of the CDDG during the clinical evaluation requires the same (or even less) time compared to their ordinary practice. Finally, the CDDG can provide a useful guidance for distinguishing disorders from normality and from other mental disorders. In fact, the CDDG have been developed with the aim to describe the essential features of each disorder, in terms of symptoms and signs usually identified by clinicians in their ordinary practice. On the other hand, cut-off and duration criteria have been avoided, unless these criteria are supported by strong scientific evidence [3, 35]. This decision has been guided by the need to develop guidelines as much as possible similar to the real-world clinical practice. The positive results of these field trials confirm that this goal has been achieved.

The overall positive ratings at the core clinical utility questions confirm that the goals of improving ease to use, goodness of fit, clarity and understandability of the ICD-11 have been fulfilled. In fact, improving the clinical utility of a classification system has been prioritized by the WHO as one of the main expected outcomes of this revised version of the ICD, in order to enhance the ability of clinicians to use the classification, to make appropriate diagnosis and provide adequate treatments [4, 40].

The implementation of the Global Clinical Practice Network (https://gcp.network) could have contributed to the positive ratings of the core questions of clinical utility [15, 16], since the diagnostic criteria had already been tested in those global, multilingual internet-based study and refined on that basis. Lastly, the ICD-11 CDDG have been conceptualized considering the most updated scientific evidences and developed with a coherent and organized structure, which are the main features of a classification system for being clinically useful [40].

Although being positive, clinicians considered the guidelines less useful for treatment selection, assessment of patients’ prognosis, communication with other health professionals and education of patients and their relatives about their condition. This finding is in line with the field trials carried out for the development of ICD-10, DSM-IV, and DSM-5 [3] and could be due to the fact that many psychiatric treatments are trans-nosographic and are not guided only by patients’ diagnosis [21, 44]. Nevertheless, clinicians’ ratings on the usefulness of the ICD-11 diagnostic guidelines for treatment selection, prognosis, and communicating with patients and families were substantially higher compared to those reported by clinicians using the ICD-10, the DSM-IV or the DSM-5 [3]. However, it is worth noting that the ICD-11 CDDG have not been developed with the primary aim to select a treatment and to assess patients’ prognosis, and the ICD-11 is not intended to be a treatment guidance [4].

In line with the WHO conceptualization of clinical utility, which cannot be considered as defined only by clinicians’ preference ratings. Other factors should be taken into account, including diagnostic reliability, scientific evidences and disease conceptualization made by clinicians in their usual practice. This conceptualization is confirmed by our findings, which showed that diagnostic validity and clinical utility are strongly correlated. In fact, the slightly lower kappa value found for schizoaffective disorder correspond to slightly lower ratings to the core clinical utility questions for the same diagnosis, compared to the diagnosis of schizophrenia. Moreover, field trial carried out in Italy confirmed the improvement of the clinical utility of the diagnosis of schizoaffective disorder, which was one of the main aims of this revision, since the clinical utility of the diagnosis of schizoaffective disorder in the ICD-10 was limited [21, 24, 33], in line with findings from the global international sample [30, 39]. This improvement can be due to the fact that the CDDG are mainly focused on the current episode rather than on the longitudinal presentation of the illness [10], supporting clinicians in making the diagnosis [7].

As possible limitations of the study, it must be noted that the ICD field trials have been carried out mainly in academic settings, in which participating clinicians could have specific research interests, with possible cognitive bias and a social desirability element influencing their responses. It is possible that clinicians not participating in this type of study will greet the ICD-11 guidelines with less enthusiasm when asked to implement them in their ordinary clinical practice. However, this limitation does not change the overall interpretation of the results, since it would be the same for any parallel assessment of clinical utility such. Another possible limitation is that the ICD-11 field trial has been carried out in only one Italian centre, thus limiting the generalization of results. However, the international design of the study and the higher number of assessment included in the global sample has mitigated this limitation.

## Conclusions

Several substantive changes have been made with the 11th revision of the Mental, Behavioural and Neurodevelopmental Disorders chapter of the ICD. Our findings show that the ICD-11 could be used in ordinary clinical settings increasing the clinical utility and validity of the diagnostic system. Therefore, we should be optimistic about the positive impact of the ICD-11 on the diagnostic skills and therapeutic management of clinicians worldwide. Once approved by the WHO General Assembly the ICD-11 will be translated into the different languages and disseminated in the WHO countries.

## Data Availability

The datasets used and/or analysed during the current study are available from the corresponding author on reasonable request.
